# Evidenzbasierte Arzneimittelversorgung bei Seltenen Erkrankungen: die Rolle der Digitalisierung

**DOI:** 10.1007/s00103-022-03605-z

**Published:** 2022-10-20

**Authors:** Frauke Naumann-Winter, Thomas Kaiser, Antje Behring

**Affiliations:** 1 Fachgebietsleitung Arzneimittel für Kinder und seltene Erkrankungen, Bundesinstitut für Arzneimittel und Medizinprodukte (BfArM), Bonn, Deutschland; 2 Institut für Qualität und Wirtschaftlichkeit im Gesundheitswesen (IQWiG), Ressortleitung Arzneimittelbewertung, Köln, Deutschland; 3 Abteilung Arzneimittel, Gemeinsamer Bundesausschuss (G‑BA), Berlin, Deutschland

**Keywords:** Arzneimittel für Seltene Erkrankungen (*Orphan Drugs*), Zulassung, Zusatznutzen, Digitalisierung, Register, Orphan drugs, Drug regulation, Added value, Digitalisation, Registry

## Abstract

Bei der Wissensgenerierung im Bereich der Arzneimittelentwicklung für Menschen mit Seltenen Erkrankungen (SE) sind besondere Schwierigkeiten zu überwinden. Welche Verbesserungen durch eine zunehmende Digitalisierung erwartet werden, wird in diesem Beitrag aus der Perspektive von 3 Institutionen im Gesundheitswesen aufgezeigt: dem Bundesinstitut für Arzneimittel und Medizinprodukte, dem Institut für Qualität und Wirtschaftlichkeit im Gesundheitswesen und dem Gemeinsamen Bundesausschuss.

Zunächst wird das Potenzial der Digitalisierung vorgestellt, auch durch eine frühere Zusammenarbeit aller Beteiligten die Effizienz der klinischen Entwicklung und der regulatorischen Entscheidungsprozesse zu erhöhen. Im Anschluss wird argumentiert, dass mit Hilfe der Digitalisierung Hürden bei der Durchführung versorgungsnaher, auch registerbasierter randomisiert kontrollierter Studien abgebaut werden sollten. Hochwertige Registerstudien sollten nicht erst nach der Zulassung, sondern bereits während des Zulassungsprozesses begonnen werden, damit die für Therapieentscheidungen notwendige Evidenz zeitnah nach Zulassung vorliegt. Abschließend wird festgestellt, dass die Verbesserung der Datenlage durch qualitative Verbesserung der Datenquellen und deren Vernetzung unmittelbar den Patient*innen zugutekommt. Verwertbare Evidenz, die über einen längeren Zeitraum – auch über die Zulassung hinaus – generiert werden kann und geeignet ist, in Entscheidungen für das Gesundheitssystem einzufließen, stellt eine effektive Arzneimittelversorgung sicher.

Die Institutionen sind sich einig, dass qualitativ hochwertige Indikationsregister als produktunabhängige, stehende Infrastrukturen entwickelt werden sollten, damit bereits früh in der Entwicklung von Arzneimitteln für SE auf hochwertige Daten zurückgegriffen werden kann.

## Abstract (en)

Knowledge generation in the field of drug development for people with rare diseases (RDs) faces particular difficulties. This paper will show what improvements are expected from increasing digitalisation from the perspective of three healthcare institutions: the Federal Institute for Drugs and Medical Devices, the Institute for Quality and Efficiency in Health Care and the Federal Joint Committee.

First, the potential of digitalisation to increase the efficiency of clinical development and regulatory decision-making through earlier collaboration of all stakeholders is proposed. Subsequently, it is argued that digitalisation should be used to reduce barriers to the implementation of care-associated randomised controlled trials, including those based on registries. High-quality registry studies should not only be started after approval but during the approval process, so that the evidence necessary for therapy decisions is available promptly after approval. Finally, it is stated that improving the evidence base through qualitative improvement of the data sources and their linkages directly benefits patients. Usable evidence that can be generated over a longer period of time – also beyond approval – and contribute to decisions within healthcare system ensures effective drug provision.

The institutions agree that high-quality indication registries should be developed as product-independent, standing infrastructures so that high-quality data can be accessed early in the development of medicines for RD.

## Einleitung

Bei der Erforschung Seltener Erkrankungen sind besondere Hindernisse zu überwinden. Es gibt bei den einzelnen Erkrankungen meist nur sehr wenige Patient*innen, die sich zur Behandlung und Nachsorge in geographisch verteilte Expertenzentren begeben. Nicht in jedem (Bundes‑)Land sind die entsprechenden Einrichtungen vorhanden, manche sind für Patient*innen nicht erreichbar. So kann sowohl die geringe Zahl der in Frage kommenden Patient*innen und je nach Erkrankung auch die erforderliche Beobachtungsdauer die Machbarkeit einer Studie erschweren. Zudem eignen sich nicht alle diagnostizierten Patient*innen gleichermaßen für die Untersuchung eines bestimmten Behandlungsansatzes. Trotz der genannten Schwierigkeiten beruht mehr als die Hälfte der Zulassungen von Arzneimitteln für Seltene Erkrankungen (Orphan Drugs) auf randomisierten klinischen Studien (RCTs), die auch weiterhin als der Goldstandard für den Erkenntnisgewinn gelten [1, 2].

Überall und jederzeit werden neu diagnostizierte und prävalente Patient*innen ihr gesamtes Leben lang medizinisch versorgt. Angesichts der vielen Fragen, die es für eine optimale Behandlung mit einem bekannten oder neuen Therapieansatz zu beantworten gäbe, haben die während der Versorgung erhobenen bzw. erhebbaren Daten sehr großes Potenzial, das Wissen über – und damit auch Behandlungsansätze für – die oft unverstandenen Seltenen Erkrankungen (SE) zu verbessern. Viele SE werden in der aktuell verwendeten internationalen statistischen Klassifikation der Krankheiten Version 10 (ICD‑10) in Überbegriffen zusammengeführt. Ihre Sichtbarkeit soll sich durch die Einführung von ICD-11 und im Übergang durch die Verwendung der *ORPHAcodes* verbessern [3]. Versorgungsnah erhobene Gesundheitsdaten, sogenannte Real-World-Daten, haben das Potenzial, Ergebnisse aus der klinischen Forschung wertvoll zu ergänzen, solange relevante Daten adäquat erhoben werden [4]. Adäquat bedeutet hier – auch in Abhängigkeit von der konkreten Fragestellung – eine verlässliche, vollständige und nachvollziehbare Datenerhebung im Rahmen eines angemessenen Studiendesigns. Dazu gehört auch die methodisch sachgerechte Auswertung der Daten.

Im Folgenden wird aus der Perspektive dreier verschiedener Institutionen im Gesundheitswesen beleuchtet, wie mit Hilfe der Digitalisierung die Wissensgenerierung im Bereich der Arzneimittelentwicklung für Menschen mit SE verbessert werden kann. Dabei wird der Beitrag der Digitalisierung insbesondere unter dem Aspekt der Qualitätsverbesserung in Bezug auf Informationsvernetzung, Datenerhebung und -auswertung betrachtet. Die 3 Institutionen sind: das Bundesinstitut für Arzneimittel und Medizinprodukte (BfArM), das ggf. über die europäische Zulassungsbehörde (European Medicines Agency) für die Zulassung von Arzneimitteln zuständig ist, das Institut für Qualität und Wirtschaftlichkeit im Gesundheitswesen (IQWiG), das evidenzbasierte Nutzenbewertungen von Arzneimitteln erstellt, und der Gemeinsame Bundesausschuss (G-BA), welcher Richtlinien über die Verordnung von Arzneimitteln erstellt.

## Position des Bundesinstituts für Arzneimittel und Medizinprodukte (BfArM)

### Flexibilität in Studiendesigns und Endpunkten bei Seltenen Erkrankungen

Erfolgreiche Arzneimittelentwicklungen beruhen gerade bei komplexen Erkrankungen auf einem guten Verständnis der zugrunde liegenden Pathophysiologie und der zu prüfenden Therapie. Sind erst Behandlungsansätze bekannt, sind selbst bei sehr seltenen Erkrankungen eine hohe Forschungsintensität und parallele Studienprogramme möglich. Neben RCTs werden bei SE auch andere Studiendesigns akzeptiert, wie z. B. einarmige Studien oder intraindividuell kontrollierte Studien [5, 6]. Das Abweichen vom Goldstandard kann durch die Seltenheit einer Erkrankung oder deren schlechte Prognose gerechtfertigt sein [7].

Studiendesigns, bei denen frühe und spätere klinische Phasen ineinander übergehen, haben in den letzten Jahren an Bedeutung gewonnen [8]. Neben Endpunkten zur Mortalität und Morbidität werden bei SE auch intermediäre Endpunkte herangezogen. Gerade in Therapiegebieten, für die es keine etablierten Endpunkte für die Messung des Therapieansprechens gibt, empfiehlt es sich, geeignete Endpunkte im Rahmen einer wissenschaftlichen Beratung im Vorfeld abzustimmen. Idealerweise werden hierfür auch Patienten(‑Organisationen) einbezogen, damit möglichst alltagsrelevante Endpunkte ausgewählt werden [9]. Bilden Endpunkte verschiedene, voneinander unabhängige Aspekte des Krankheitsverlaufs ab, ist es zudem möglich, einen Gesamteindruck von der Wirksamkeit zu gewinnen, was gerade bei der Interpretation von Studien mit sehr kleinen Fallzahlen hilfreich sein kann.

Harte klinische Endpunkte werden für Zulassungen zwar regelmäßig erhoben, treten aber ggf. zu selten auf, um robuste Aussagen treffen zu können, oder sie können durch das Studiendesign oder durch sich anschließende Therapielinien nicht allein dem primär untersuchten Medikament zugeordnet werden. Häufig ist die therapeutische Indikation im Rahmen der Erstzulassung auf eine bestimmte Population eingeschränkt, die nur einen Teil der Patient*innen in der klinischen Praxis darstellt. Es liegt in der Verantwortung der Zulassungsinhaber, nachfolgende Indikationserweiterungen zu beantragen. Wenn es mehrere Untergruppen mit einem sich relevant unterscheidenden Krankheitsverlauf gibt (z. B. verschiedene Altersgruppen), können klinische Studien nicht für alle Untergruppen aussagekräftig auswertbar geplant werden – zumindest nicht vor der Erstzulassung.

### Versorgungsdaten für die Zulassung?

In der Routineversorgung wird die Wirksamkeit einer bestimmten Substanz selten systematisch quantifiziert bzw. sind entsprechende Informationen aufgrund der fehlenden Standardisierung der Kriterien bzw. Erhebungsmethodik [10] meist nicht valide. Real-World-Daten sind bisher nur in Ausnahmefällen zulassungsbegründend, auch bei Indikationserweiterungen [11]. Für eine Zulassung ist aber auch die konkrete Exposition relevant, damit die Bedingungen für ein günstiges Nutzen-Risiko-Verhältnis bestimmt werden können (z. B. Kontraindikationen, Warnhinweise). Reine Routinedaten über Verschreibungen oder Abgabe bilden nicht zuverlässig die tatsächlich applizierte oder von den Patient*innen eingenommene Dosis ab, sobald wegen z. B. individueller Verträglichkeit oder Adhärenz von der zugelassenen Dosierung abgewichen wird.

Sekundärdaten können Studiendaten wertvoll ergänzen, v. a. wenn es um Sicherheitsaspekte oder Langzeiteffekte geht. In Deutschland wird derzeit das Forschungsdatenzentrum Gesundheit am BfArM aufgebaut, in dem Abrechnungsdaten aller gesetzlich Krankenversicherten und von den Versicherten selbst freigegebene Daten der elektronischen Patientenakten auf Antrag an Nutzungsberechtigte in gesicherten Analyseumgebungen bereitgestellt werden [12]. Im Verlauf ist geplant, die Datenzentren auf europäischer Ebene zu vernetzen und die sekundäre Nutzung von Versorgungsdaten voranzubringen [13, 14].

### Daten aus forschungsorientierten Registern sind vielversprechend

Die Detailtiefe hinsichtlich der Grundinformationen und des Krankheitsverlaufs von mehr oder weniger selektiv eingeschlossenen Patient*innen kann sich zwischen Registern erheblich unterscheiden, so dass in jedem Einzelfall geprüft werden muss, ob sich ein Register für bestimmte Fragestellungen eignet. Die Qualitätsanforderungen für ein Register, das für den Wissensgewinn geeignete ist, sind aufgrund des hohen Verzerrungspotenzials sehr hoch [15].

Register können sowohl im Vorfeld wertvolle Daten zur Studienplanung beisteuern als auch für den Zeitraum nach der Zulassung beauflagt werden, um konkrete Unsicherheiten im Nutzen-Risiko-Verhältnis zu adressieren [16]. Das Wissen um den natürlichen Krankheitsverlauf kann u. a. eine valide Fallzahlplanung und die Festlegung einer geeignete Studiendauer ermöglichen bzw. kann es die Grundlage für die Erhebung gängiger Behandlungsmuster oder Ereignisraten in Untergruppen bilden und ggf. die Extrapolation von Studienergebnissen begründen. Systematisch, vollständig und qualitätsgesicherte Daten aus der Versorgung könnten dazu beitragen, den Surrogatcharakter eines Biomarkers näher zu beleuchten. Der Aufwand für die Gründung eines Registers oder auch nur die Planung einer effizienten Studie in einem bestehenden Register sollte jedoch nicht unterschätzt werden.

Die 2017 gegründeten *European Reference Networks* (ERN), in denen die Expertise bzgl. bestimmter seltener Therapiegebiete vernetzt wird, könnten besonders dazu beitragen, den Fokus auf Therapiegebiete zu lenken, für die es bisher keine Behandlungsansätze gibt [15, 17, 18]. In Universitäten werden oft die Grundlagen der innovativen Arzneimittelentwicklung für komplexe Erkrankungen geschaffen. Dass die Förderung der ERN durch die Europäische Kommission die Standardisierung und Verbesserung der Datenqualität sowie die Nachhaltigkeit des Datenzugangs von Krankheitsregistern beinhaltet, ist deshalb vielversprechend [19]. Patient*innen mit SE sind zudem bekannt für ihre überdurchschnittliche Bereitschaft, ihre Daten für die Forschung zur Verfügung zu stellen [20]. Diese Bereitschaft könnte auch die Weiterentwicklung der sogenannten Wearables (am Körper getragene Minicomputer), die kontinuierlich Daten aus dem Alltag erheben, befördern [21]. SE könnten somit ein Vorreiter beim Erkenntnisgewinn aus Daten aus der Versorgung werden.

Angesichts der ständigen Verbesserungen der Datenlage, auch durch die geplanten Verknüpfungen mit weiteren Datenbanken, empfiehlt es sich, verfügbare Sekundärdaten hinsichtlich ihrer Eignung für die Beantwortung aktueller Fragestellungen kritisch zu prüfen. Überdies kann eine studienbegleitende prospektive Datensammlung auch vor Markteintritt des Produktes wertvolle Daten liefern. Die Rekrutierung von Patient*innen in Zulassungsstudien kann über bereits bestehende Register erleichtert werden, wenn z. B. die im Register erhobenen Daten in eine nachfolgende Studie einfließen (anstelle einer *Run-in-*Phase vor der Behandlung).

Eine für den Erkenntnisgewinn besonders kostbare Zeit ist die, wenn erste translatierbare Ansätze für die Behandlung einer Erkrankung gefunden wurden. Die Aussicht auf ein möglicherweise bald zur Verfügung stehendes Arzneimittel setzt ein Schlaglicht auf die jeweilige Erkrankung. Gerade bei extrem seltenen Erkrankungen, bei denen Problemkonstellationen zwischen Zulassung und Health Technology Assessment (HTA) bereits früh absehbar sind, sollte die bereits bestehende Zusammenarbeit im Rahmen der wissenschaftlichen Beratung zwischen Zulassung und HTA intensiviert werden [22].

## Position des Instituts für Qualität und Wirtschaftlichkeit im Gesundheitswesen (IQWiG)

### Orphan Drugs: die Evidenz für Therapieentscheidungen ist oft unzureichend

In Deutschland können neue Arzneimittel, auch Orphan Drugs (Arzneimittel für SE), direkt nach der Zulassung eingeführt und erstattet werden, ohne dass dies einer vorhergehenden Bewertung (sogenannte vierte Hürde) bedarf [23]. Deutlich über 90 % der zugelassenen Orphan Drugs sind daher in Deutschland im Rahmen der gesetzlichen Krankenversicherung (GKV) verfügbar [23].

Eine aktuelle Analyse des IQWiG zeigt, dass für einen Großteil der Orphan Drugs zum Zeitpunkt ihres Marktzugangs in Deutschland keine Evidenz für einen Zusatznutzen gegenüber Therapiealternativen vorliegt: In mehr als der Hälfte der Fälle lautete die Schlussfolgerung des G‑BA bislang: „Kein Beleg für einen Zusatznutzen“ [24]. Zu einem ähnlichen Ergebnis kommt das Patented Medicine Prices Review Board (PMPRB) bei der Bewertung von hochpreisigen Orphan Drugs für das kanadische Gesundheitssystem im Zeitraum 2010–2019 [25].

Zumeist beruhte die Feststellung eines nicht belegten Zusatznutzens jedoch nicht auf hochwertigen, vergleichenden Studien, sondern war das Ergebnis der unzureichenden Evidenzbasis, also insgesamt fehlender Studien [24]. In der Konsequenz bedeutet dies: In mehr als der Hälfte der Fälle stehen bei Markteinführung keine ausreichenden Informationen für die Therapieentscheidung zur Verfügung und es ist daher unklar, ob ein neues Orphan Drug besser, gleich gut oder schlechter als die zur Verfügung stehenden Therapieoptionen abschneidet. Wie kann Digitalisierung dabei helfen, diesen Evidenzmangel zu beheben?

### Digitalisierung und versorgungsnahe Daten – kein Selbstzweck

In einem aktuellen White Paper des US-amerikanischen Institute for Clinical and Economic Review (ICER) wird ebenfalls die Notwendigkeit einer Verbesserung der Evidenzbasis für Orphan Drugs konstatiert [26]. Dabei werden einerseits im Zusammenhang mit der Digitalisierung Voraussetzungen für die Nutzbarkeit sogenannter Real World Evidence (RWE) beschrieben, z. B. die Erfassung patientenberichteter Endpunkte (PROs), die Weiterentwicklung der ICD-10-Kodierung zur granulären Erfassung von SE sowie die verpflichtende Dokumentation in Registern im Falle einer Erstattung eines Orphan Drug. Um dies zu erreichen, wird u. a. vorgeschlagen, hochwertige Register und die Zusammenführung verlässlicher Datenquellen zu fördern. Gleichzeitig wird jedoch davor gewarnt, dass die zunehmende Verschiebung der Evidenzgenerierung auf einen Zeitpunkt nach Zulassung mittels RWE die Evidenzbasis sogar verschlechtern kann:

„RWE as a policy option, taken to an extreme, might further undermine efforts to establish reasonable requirements for randomized trials, or for trials of adequate duration and comprehensiveness to understand the risks and benefits of an emerging treatment“ [26].

Bei der Frage, wie Digitalisierung die Evidenzbasis für Orphan Drugs stärken kann, sind daher insbesondere 2 Punkte von entscheidender Bedeutung:

- Digitalisierung sollte dazu beitragen, die organisatorischen und finanziellen Hürden bei der Durchführung hochwertiger, versorgungsnaher Studien bei Orphan Drugs abzubauen. Dazu gehören insbesondere registerbasierte RCTs (RRCTs).
- Hochwertige Evidenz sollte nicht erst nach der Zulassung, sondern bereits während des Zulassungsprozesses generiert werden, damit die für Therapieentscheidungen notwendige Evidenz zum Zeitpunkt des Marktzugangs, mindestens aber verlässlich kurz danach, vorliegt.

Beide Punkte sind kein Widerspruch dazu, dass der Wert versorgungsnaher Daten in den letzten Jahren zunehmend hervorgehoben wird, im Gegenteil: Prinzipiell kann mit RRCTs schnell, versorgungsnah und ergebnissicher die für die Therapieentscheidung notwendige Evidenz generiert werden [27–29]. Solche Studien auf Basis einer bereits stehenden Dateninfrastruktur (Register) sind überdies besonders kostengünstig [30].

### Hochwertige Forschung mit versorgungsnahen Daten gestalten, nicht verhindern

Für dieses Verständnis ist es jedoch notwendig, die Begriffe „Real World Data“ (RWD) und „Real World Evidence“ (RWE) nicht fälschlicherweise mit nichtrandomisierten Studien gleichzusetzen, was national und international zunehmend erkannt wird [31, 32]. Es ist daher bedauerlich, dass im Rahmen des europäischen Datenraums „DARWIN EU“ (Data Analysis and Real World Interrogation Network) nur nichtinterventionelle Studien vorgesehen sind [14]. Diese Beschränkung ist zum einen nicht kongruent mit der von der Europäischen Arzneimittel-Agentur (EMA) publizierten Leitlinie zur Durchführung registerbasierter Studien: In dieser werden RRCTs als Option vorgesehen [15]. Zum anderen festigt diese Beschränkung von DARWIN EU genau das, wovor im ICER-Bericht gewarnt wird und was derzeit bei der Evidenzbewertung für die Versorgung von Patient*innen sichtbar wird: den Mangel an aussagekräftigen, vergleichenden Daten zur Unterstützung evidenzbasierter Entscheidungen für Menschen mit SE. Digitalisierung und versorgungsnahe Daten sind jedoch nicht per se hilfreich, sie müssen sinnvoll eingesetzt werden, damit die Evidenzbasis für Therapieentscheidungen wirklich verbessert wird.

Die Fokussierung auf qualitativ hochwertige Studien, die Therapieentscheidungen unterstützen, ist im Übrigen gerade bei SE aufgrund der geringeren Fallzahl sinnvoll. Denn bei nichtrandomisierten vergleichenden Studien ist die Datenanforderung in der Regel höher und nicht geringer als bei RCTs. Dies gilt sowohl für den Datensatz selbst (Erhebung relevanter Confounder (Störgrößen)) als auch für die Fallzahl. Denn die Fallzahl muss bei nichtrandomisierten Vergleichen groß genug sein, um überhaupt methodisch sinnvoll für Confounder adjustieren zu können, und der beobachtete Effekt muss groß genug sein, um aufgrund des unbekannten Confounding mit ausreichender Sicherheit einen Vorteil einer Therapieoption ableiten zu können [33]. Dass RCTs prinzipiell auch bei seltenen und sehr seltenen Erkrankungen durchführbar sind, wurde vielfach nachgewiesen [34–36]. Viele dieser RCTs untersuchen derzeit jedoch nicht die versorgungsrelevante Frage eines Therapievergleichs mit relevanten Therapiealternativen [24, 36].

### Beschleunigte Evidenzgenerierung – Ein Vorschlag zur Verbesserung der Versorgung

Wenn beim Thema Arzneimittelversorgung von „Beschleunigung“ die Rede ist, dann ist damit zumeist die Beschleunigung der Zulassung mit dem Ziel des beschleunigten Zugangs zu Orphan Drugs gemeint [37]. Die sich daraus ergebenden Defizite fehlender Evidenz für Therapieentscheidungen bei Marktzugang sind jedoch empirisch nachgewiesen [24, 25]. Und auch das Versprechen einer Evidenzgenerierung nach Marktzugang [38] hat sich nicht erfüllt, auch Jahre später liegen zumeist keine versorgungsrelevanten vergleichenden Studien vor [24]. Aus regulatorischer Sicht sind die bisherigen Erfahrungen mit RWD offenbar eher ernüchternd [39, 40].

Aus Sicht des IQWiG spricht nichts gegen eine beschleunigte evidenzbasierte Versorgung, aber es spricht sehr viel gegen eine beschleunigte Zulassung ohne relevante Evidenz für die Versorgung.

Es ist daher notwendig, mit Hilfe der Digitalisierung zum Zwecke der Evidenzgenerierung für Orphan Drugs eine hochwertige Datenplattform aufzubauen und diese für die Evidenzgenerierung bereits vor Zulassung zu nutzen. Dazu gehört auch, dass organisatorische und finanzielle Hürden zur Nutzung dieser Datenplattform für interventionelle, versorgungsnahe Studien (z. B. RRCTs) abgebaut werden, also eine entsprechende Forschungskultur unterstützt wird. Die Datenplattform muss, ausgestattet mit einem Basisdatensatz, fragestellungsbezogen erweiterbar sein, um die unterschiedlichen Informationsbedarfe für die verschiedenen Erkrankungen abzudecken. Idealerweise sollten bereits die Zulassungsstudien unter Verwendung dieser Datenstruktur durchgeführt werden, um die Langzeitnachbeobachtung nach Abschluss der ursprünglichen Studie zu ermöglichen. Vergleichende Studien sollten, unabhängig von den Anforderungen an die Zulassung, noch während des Zulassungsverfahrens begonnen und vor Erteilung der Zulassung vollständig rekrutiert werden.

Mit einer solchen Datenplattform, verbunden mit der Etablierung einer Forschungskultur zur Durchführung hochwertiger versorgungsnaher Studien, könnten wir die sichtbaren Evidenzlücken schließen und für noch mehr Menschen mit SE das erreichen, was ursprünglich beabsichtigt war: „Patienten mit seltenen Leiden müssen dasselbe Recht auf gute Behandlung haben wie andere Patienten“ [41].

## Position des Gemeinsamen Bundesausschusses (G-BA)

### Die frühe Nutzenbewertung und Evidenzanforderungen bei Orphan Drugs

Bei der frühen Nutzenbewertung nach § 35a Fünftes Buch Sozialgesetzbuch (SGB V) beurteilt der G‑BA anhand der vorgelegten Evidenz und unter Berücksichtigung des Versorgungskontextes, mit welcher Wahrscheinlichkeit und in welchem Ausmaß ein Zusatznutzen im Vergleich zu einer vom G‑BA zuvor bestimmten zweckmäßigen Vergleichstherapie (zVT) vorliegt [42]. Auf Basis dieser Feststellungen wird der Erstattungsbetrag für die Arzneimittel verhandelt. Für Orphan Drugs gelten die besonderen Regeln, dass in der initialen Bewertung ein Zusatznutzen aufgrund der Zulassung als belegt gilt und ein Vergleich gegenüber einer zVT nicht gefordert ist.

Bei der Nutzenbewertung von Orphan Drugs steht der G‑BA vor der besonderen Herausforderung, dass graduierte Feststellungen zum Zusatznutzen häufig ohne Vergleich und auf Basis einer sehr unsicheren Erkenntnislage getroffen werden müssen. Nur bei etwa einem Drittel der Orphan Drugs liegen randomisierte kontrollierte Vergleichsstudien – häufig ausschließlich Placebovergleiche – vor. Da in den klinischen Studien für Orphan Drugs die patientenrelevanten Endpunkte zum Teil noch nicht erreicht bzw. nicht erhoben wurden oder Ergebnisse nur für eine eingeschränkte Patientenpopulation gezeigt werden konnten, werden häufig ersatzweise klinische Surrogatparameter herangezogen.

Es ist festzustellen, dass identifizierte Evidenzlücken z. B. in Bezug auf einen fehlenden Vergleich oder fehlende Ergebnisse zu patientenrelevanten Endpunkten, in mehr als 50 % der Fälle auch nicht für solche Orphan Drugs geschlossen werden konnten, für die nach einigen Jahren eine Neubewertung im Rahmen einer regulären Nutzenbewertung erforderlich ist [24]. Auch wenn dies nicht allein ein Orphan-Drug-Problem ist [43], so sollte es insbesondere aufgrund der unzureichenden Kenntnislage bei SE ein Anliegen sein, Daten zu den entsprechenden Therapien weiter zu erheben. Digitalisierung kann hier zu einer besseren Vernetzung der Datenquellen und zu einer strukturierten, nahezu vollständigen Datenerhebung mittels Indikationsregister beitragen.

Um weitere Evidenzgenerierung bei Orphan Drugs nach der Zulassung zu fördern und zu initiieren, hat der G‑BA die Möglichkeit, eine anwendungsbegleitende Datenerhebung (AbD) zu fordern [44].

### Erfahrungen aus der Nutzenbewertung mit Registerdaten von Orphan Drugs

Derzeit eignen sich Registerstudien am besten, um Daten anwendungsbegleitend zu erheben. Sie wurden bereits vor der entsprechenden Gesetzesänderung im „Gesetz für mehr Sicherheit in der Arzneimittelversorgung“ (GSAV) vom G‑BA bei einigen Orphan Drugs gefordert [45–47], bei denen die EMA die pharmazeutischen Unternehmer beauflagte prospektive, langfristige internationale Beobachtungsregister einzurichten, um z. B. Informationen über die Epidemiologie der Krankheit, klinische Ergebnisse zur Sicherheit, Wirksamkeit und z. T. zur Lebensqualität zu sammeln. Auch wenn entsprechende Registerstrukturen geschaffen wurden, kann festgestellt werden, dass der methodische Umgang mit solchen Datenerhebungen und Auswertungen zum Zwecke der Nutzenbewertung noch nicht geübt ist [48].

Damit eine anwendungsbegleitende Datenerhebung für die Nutzenbewertung verwertbar ist, sollten von allen Beteiligten Anstrengungen unternommen werden, eine gleichförmige, vollständige Dokumentation der relevanten Endpunkte für alle Patient*innen sicherzustellen, unabhängig von deren Therapie. Ansonsten sind die Datensätze bereits durch die Unterschiedlichkeit und Unvollständigkeit der Dokumentation verzerrt und nicht auswertbar. Um die Vorteile der Digitalisierung und strukturierten Dateneingabe zu nutzen, sind Vorgaben in Registerprotokollen zur Standardisierung der Datenerfassungen sowie zu Art und Umfang einer Informationsübertragung, z. B. aus Krankenakten, erforderlich. Beispielsweise können Confounder-Analysen in Bezug auf Patientencharakteristika ohne Standardisierung und einheitliche Operationalisierung der Begrifflichkeiten für alle Anwender im Register nicht bewertet und ausgewertet werden. Neben der Planung eines Registers selbst sind die Registerstudien und deren statistische Auswertungen – analog zum Aufsetzen anderer klinischer Studien – mittels Protokoll zu planen. Die Erstellung eines statistischen Analyseplans in Kenntnis der Ergebnisse der Registerdaten stellt eine Unabhängigkeit der Auswertungen in Frage.

### Lösung für die Nutzenbewertung durch Digitalisierung

Auf europäischer Ebene wird bereits durch verschiedene Projekte an den technischen Voraussetzungen, wie einer einheitlichen Nomenklatur sowie verbindlichen IT-Standards und einheitlichen Datenaustauschformaten, zur besseren Interoperabilität gearbeitet. Jedoch ist die hohe Qualität der digitalen Daten eine Grundvoraussetzung, damit diese als valide Entscheidungsgrundlage dienen können. In besonderem Maße gilt dies bei SE, da jeder fehlende Datensatz erhebliche Auswirkungen auf die Ergebnisse und deren Verlässlichkeit hat. Der Anspruch ist mehr darauf ausgerichtet, qualitativ hochwertige prospektive Daten zu erhalten, und weniger auf das Sammeln möglichst vieler Daten für eine retrospektive Nachlese.

Die derzeitigen Routinedaten in Deutschland, wie Abrechnungsdaten oder die Daten, die mit der elektronischen Patientenakte verfügbar sein werden, sind aktuell nicht hinreichend entwickelt, um auf einfache Weise Therapievergleiche zu ermöglichen. Die Datensätze sind derzeit zu unvollständig (z. B. aufgrund der Grenze zwischen stationärer und ambulanter Versorgung), zu fehlerhaft (auch aufgrund möglicher Abrechnungsvorgaben) und für SE zu undifferenziert, um für die Nutzenbewertung von Arzneimitteln geeignet zu sein. Registerbasierte Studien sind deshalb für die Nutzenbewertung (vorerst) der einzige Weg, die Datenlage zu verbessern, sofern dies nach der Zulassung erforderlich ist. Wünschenswert wäre es, vor der Zulassung mit Studien oder Registern zu beginnen, die nach der Marktzulassung weitergeführt werden können. Dann ließe sich bei unsicherer Evidenzlage nach der Zulassung eine ergebnisoffene Forschung weitestgehend unbeeinflusst von Rekrutierungsproblemen für den Vergleichsarm durchführen.

Diese Studien könnten der Beginn von modular erweiterbaren Indikationsregistern sein, sofern weitere Interventionen untersucht werden sollen. Die Ergebnisse dieser Studien müssen zudem öffentlich zugänglich und digital abrufbar sein, um darauf aufbauende Auswertungen und Untersuchungen zu unterstützen.

Die bisherigen Erfahrungen haben gezeigt, dass, selbst wenn ein gutes Register bereits vorhanden ist, die Konzipierung einer Registerstudie einen erheblichen Planungs- und Zeitaufwand bedeutet. Es besteht deshalb die Gefahr, dass nach der Markteinführung eines Arzneimittels wertvolle Zeit verloren geht, in der noch ein hohes Interesse an der Beantwortung ungeklärter Fragen zu dem Arzneimittel vorhanden ist. Der Aufwand an Kosten und Ressourcen und die Erwartungen aller Beteiligten an die zu generierenden Erkenntnisse sind so erheblich, dass wir es uns nicht leisten sollten, auf schlechter Datenqualität gründende Entscheidungen zu treffen. Dennoch muss die vorhandene Evidenz insbesondere bei Orphan Drugs mit Augenmaß bewertet werden.

## Fazit

Die vorhergehenden Ausführungen zeigen einerseits, dass unterschiedliche Institutionen im Gesundheitswesen durchaus unterschiedliche Sichtweisen auf die Frage nach der geeigneten Art und Weise der Wissensgenerierung bei SE haben. Dies ist auch durch die unterschiedlichen Aufgaben der beteiligten Institutionen bedingt. Während mit der Zulassungsentscheidung (BfArM bzw. EMA) ein Marktzugang wirksamer und sicherer Arzneimittel ermöglicht wird, wird in der vergleichenden Nutzenbewertung (IQWiG/G-BA) zum einen der Stellenwert eines neuen Wirkstoffs in der Versorgung bewertet und zum anderen die Grundlage für Verhandlungen über den von der Versichertengemeinschaft zu tragenden Preis für das Arzneimittel gebildet. Diese unterschiedlichen Sichtweisen werden im vorliegenden Artikel bewusst offengelegt. Denn für die Weiterentwicklung der Forschungslandschaft mit Hilfe der Digitalisierung ist es wichtig, diese unterschiedlichen Perspektiven zu kennen, zu verstehen und zu berücksichtigen.

Andererseits lassen sich bei allen Unterschieden auch wichtige Gemeinsamkeiten der beteiligten Institutionen feststellen. Dazu gehört insbesondere die Art der Daten(‑Plattform), die mit Hilfe der Digitalisierung erreicht werden sollen: qualitativ hochwertige Indikationsregister, und zwar als stehende Infrastrukturen, damit bereits früh in der Entwicklung von Arzneimitteln für SE auf hochwertige Daten zurückgegriffen werden kann. Dadurch kann auch die Effizienz jedes einzelnen Schrittes des oft langen Weges einer Arzneimittelentwicklung gesteigert werden. Dies ist nicht einfach umsetzbar und mit entsprechenden finanziellen wie organisatorischen Investitionen verbunden. Die ungünstigere Alternative ist jedoch, weiterhin in vielen Bereichen unwissend zu bleiben oder Wissen erst dann zu generieren, wenn es im Grunde kaum noch benötigt wird, weil schon der nächste Entwicklungsschritt ohne ausreichendes Wissen die Versorgung prägt. Es lohnt sich also, in gutes Wissen für eine bessere Versorgung zu investieren.
